# Creativity and the Trauma of COVID‐19: How do Foundation Level Art and Design Students Navigate a Liminal Journey during a Pandemic?

**DOI:** 10.1111/jade.12405

**Published:** 2022-05-08

**Authors:** Elaine Robertson, Judy Thomas, Mark Bailey

**Keywords:** art education, design education, online education, COVID‐19 pandemic, hybrid delivery

## Abstract

This article explores the impact of COVID‐19 on the physical learning spaces of art and design students to consider how this has informed and influenced the creative process, emotional resilience and engagement with learning during this difficult year of restrictions. More specifically, it draws on the experiences of students in a Foundation Art and Design programme in a Further Education college in the North of England as a case study. As a transitional year between Further Education (FE) and Higher Education (HE), this programme is designed to be transformative in terms of theoretical knowledge, practical skills and shaping student identities through ‘becoming’ creative practitioners. Concepts of liminality and liminal spaces provide a lens through which to offer an account of the nature and scope of these transformative experiences over the past year. As an accidental ethnography (Levitan *et al.* 2017), aspects discussed suggest that whilst COVID‐19 had a significant negative impact on the experiences and progression of this cohort of students, there have also been many positive outcomes suggesting that this particular programme has maintained its transformative ambitions.

## Introduction

As a transitional FE programme, the Foundation Diploma Art and Design provides an opportunity to address widely recognised gaps resulting from diminishing art curriculums in British school education (Adams 2009; Page *et al*. 2006; Vaughan *et al*. 2008). The overarching aim of this programme is to engender changed mindsets by developing knowledge, practical and technical skills, and the social and collaborative skills required to support creative confidence. This year, therefore, offers a transformative experience, a ‘betwixt and between’ space (Turner 1969) through which students have departed from the familiarity and normative realms of the school environment and enter into a new ‘process of becoming’. For Foundation students, this offers new forms of creative practitioner identity across visual arts disciplines.

The transformative journey on the Foundation programme is usually highly successful in guiding and supporting visual arts students towards intended progression routes, particularly HE. However, COVID‐19 forced a very different learning journey and experience on Foundation students during 2020–21. The familiar dialogic and interactive approach to teaching and learning for visual arts faced significant interruption, with the majority of face‐to‐face studio teaching replaced by online learning. As a result, visual arts educators and students faced many challenges to maintain ongoing dialogue and collaboration. Additionally, as a practical subject, access to material resources and facilities were severely restricted. In response to this, the article reflects on how this cohort of students experienced a liminal journey over the year.

Using a cohort of Foundation Art and Design students following a British curriculum at an FE college in the North of England (NCL‐FAD hereafter) as a case study, this article reflects on tutor observations and student feedback to explore experiences over 2020–21. As an accidental ethnography (Levitan *et al*. 2017), pre‐existing data was gathered during normative pedagogical processes rather than as part of a pre‐planned research project. This allows a practitioner‐oriented, pragmatic approach, reflecting on ‘accidentally’ gathered extant data to offer insight into educational experiences. Focusing on the purpose and intent of NCL‐FAD, research considered the following questions:

• How did COVID‐19 affect the physical spaces and student engagement over the past year?
• How did students respond to this ongoing situation?
• To what extent has the programme fulfilled its purpose for this cohort of students?

Discussion considers the socio‐cultural context holistically (Lofthouse 2014) and reflectively as a contribution to *collaborative and practical ways of knowing* (Morley 2008, p. 265). Whilst some may argue the ‘insider’ approach lacks objectivity and influences judgements, this positioning affords the author unique insight from within (Kemmis *et al*. 2013). In selecting specific examples, axiological assumptions have been made which consider the range of student activities to identify and acknowledge how these have added value to the transformative learning journey.

## LIMINAL JOURNEYS AND GATEWAYS TO TRANSFORMATION

The roots of liminality and liminal spaces can be found within the early anthropological studies of Arnold van Gennep’s ‘Les rites de passage’, originally published in 1909 and subsequently re‐evaluated by Victor Turner (1969). Van Gennep observed the rituals marking a persons transition from one state or status to another identifying three stages;

1 Pre‐liminal stage (isolation or separation);
2 Liminal stage (adaptation and transition);
3 Post‐liminal stage (incorporation, internalisation and acceptance of new norms and values).

For Turner (1969), the liminal stage is very much a functional phase where an individual is released from previous states. It is a state where a person is in‐between, neither here nor there, ‘betwixt and between’, situated in the gap between past and future. Progress through this liminal stage fulfils a threshold function allowing the individual to move into an alternative reality through creative free will, therefore representing a positive transformation.

Considering transformation further, the learning process can also be explored through the framework of threshold concepts (Cousin 2006; Meyer & Land 2003), where learning leads to new levels of ability and a more developed understanding. Threshold concepts (TC) represent portals or gateways to new capabilities, yet the characteristics of these can be problematic and difficult to navigate for students. A TC should be transformative, aligning the learning undertaken to inspire alternative thinking or transformed perspectives. This new understanding should be irreversible, once understood it supports a cognitive shift in confidence. TCs should be integrative, bringing together a range of other aspects within a subject or discipline, enabling students to understand these holistically. The fourth characteristic of TCs is that learning should be bounded, framing the knowledge within a discipline, for example through the use of specialised terminology to delineate conceptual space. However, this TC has been contested as limiting, perhaps very true within the context of art and design education where the blurring of disciplinary boundaries continues to evolve. Finally, a TC often requires the acquisition of knowledge which may be troublesome for learners to grasp, perhaps alien or counter‐intuitive, and often challenging when conflicting with previous understandings.

## THE FOUNDATION MODEL AS A ‘RITE OF PASSAGE’

Positioning NCL‐FAD as a ‘rite of passage’ and transformative journey, the programme acts as a ‘bridge’ between school and HE with two key aims. First and foremost, the programme aims to develop theoretical knowledge and practical skills, therefore addressing a perceived skills gap between school‐based curriculums and the requirements of HE (Adams 2009; Page *et al*. 2006; Vaughan *et al*. 2008). Previous education for these students often follows transmissive rather than transformative approaches to learning which fail to support the development of inquiring minds, dispositions of critical and conceptual thinking, and the ability to problem solve. Each is required for a successful transition to Higher Education (HE).

Secondly, following often limited and generalised school arts programmes, students have the opportunity to progress through a range of art and design disciplines they have not previously been exposed to. This opportunity to explore new pathways whilst developing ‘creative confidence’ (Kelley & Kelley 2015), supports informed decisions on progression routes. Through these two key aims, the NCL‐FAD creates a transformative and purposeful learning journey.

Although van Gennep’s original anthropological research was based on a social model, his three stages of ritualistic ‘rite of passage’ provide an interesting sequence to offer insight into the liminal learning journey of NCL‐FAD students across all three units of the programme. During the first unit, students enter the pre‐liminal stage following a departure from the familiarities of school education and friendship groups sustained over the years. Leaving behind previous identities, they experience the isolation and separation defined in this stage. The NCL‐FAD programme begins with the opportunity to explore Fine Art, 3D Design, Visual Communication, Fashion and Textiles. This initial investigatory and diagnostic unit helps students become familiar with the range of disciplines and pathways available on the programme by working through one project in each area to develop contextual awareness. This supports an informed choice of specialist practice for subsequent units.

As students progress to the second unit, developing specialist practice, they have already begun to establish a new creative identity by adapting to new creative processes and using ambiguous terms such as originality, imagination, innovation and sophistication. This unit allows for the adaptation and transition defined in van Gennep’s liminal stage as students experience a more focused sense of ‘becoming’ a creative practitioner. In this respect, learning takes an ontological turn by linking the processes and skills to a deeper understanding of performing practice rather than simply knowing about practice. This approach responds to growing argument for a shift towards the foregrounding of ontological over epistemological approaches in higher education pedagogies (Dall'Alba & Barnacle 2008; Pollard 2014), yet offers an ideological balance between the two. Students are able to define individual creative ambitions through a better understanding of the possibilities for creative work in each area. Pathways reflect those within professional practice and allow students to narrow creative focus further through more discipline‐specific work. The practical and procedural knowledge and skills embedded within the creative process, and specific aspects within this, are often new to students. Unlearning previous ways of working often guided by clear tasks, students experience the discomfort of liminal spaces as they struggle to grasp troublesome knowledge (Cousin 2006; Meyer & Land 2003). Initially, projects take learners out of their comfort zone and into liminal spaces where uncertainty and ambiguity exist (Turner 1969). Understanding may be partially suspended as learners are typically faced with problem‐based learning scenarios (PBL) where there are no right or wrong solutions. This constructivist approach allows students to develop enquiring minds and nurture the positive effects of creative thinking (Bose *et al*. 2006; Lor 2017). While some suggest that teacher‐initiated problems are less authentic (McIntosh 2012), project work on NCL‐FAD offers a broad approach to themes and problems. Outcomes for work are not pre‐defined, leaving project briefs open to inquisitiveness, interpretation and a multitude of possible outcomes.

This approach further cements the notion of liminality, where at micro‐level each project represents a journey into the unknown. Transformation is not simply rooted in outcomes solving aesthetic problems but emerges through a deep learning engagement with the creative process. In this respect, student development can be mapped across surface and deep learning (Entwistle 1997). Where surface learning may be apparent through the remembering of practical and technical processes, a deeper engagement emerges as students begin to focus on individual concepts requiring their own ideas and responses. This second unit supports students to further develop a sense of self and ‘becoming’ as creative practitioners.

The final unit allows students to independently demonstrate new creative identities. Students assume control of their own learning and direction by independently initiating, researching, developing and realising a project of their own choice. In this sense, the unit seeks to consolidate transformative learning journeys where self‐direction and higher levels of critical self‐reflection contribute further to a growing awareness of who they are as creative practitioners. This consolidation of developed knowledge, new creative identities and changed mindset inspires self‐belief and creative confidence. A sense of ownership is encouraged throughout.

Work produced in this final unit ordinarily culminates in a physical exhibition where students embrace new identities to share their creative practice in a formalised setting. The exhibition usually attracts an audience of between 150 and 200 people during the open evening and is subsequently visited by alumni and school groups, with students acting as facilitators. This final experience for NCL‐FAD students allows them to demonstrate acceptance of new norms and values as defined in van Gennep’s post‐liminal stage, where they have begun to internalise this new sense of self and creative identity.

Following 10 years of teaching on this programme, it is clear to the author that the opportunities for growth and transformation support the development of creative confidence. Students begin the programme having previously been guided with clear and structured goals within school‐based curricula, yet emerge from the programme with an increased ability to deal with the ‘complexities of uncertainty’ (Vaughan *et al*. 2008, p. 6). This is very much a transformative programme, engendering changed mindsets and identity shifts as students begin to shape their creative practice within a particular discipline.

## WHEN CREATIVITY AND TRAUMA COLLIDE DURING A PANDEMIC

Considering the questions posed around the impact of ongoing COVID‐19 restrictions on the physical and psychological spaces over the past year and the efficacy of the programme, the following insights are based primarily on tutor observation and reflection.

### Trauma and new beginnings

When the 2020–21 cohort began their NCL‐FAD creative journey, there was already growing concern around the impact of COVID‐19 on young people’s mental health (Newlove‐Delgado *et al*. 2021). Trauma‐related, negative emotions quickly became apparent through the general demeanour of students, with clear signs of anxiety and a distinct lack of motivation, resilience and confidence. This reflects the findings of Montacute & Holt‐White (2021) who identify 87% of university students suffering from negative impacts as a result of this pandemic. Whilst this relates to university students, figures may be significantly higher for school leavers such as those on the Foundation programme. Having been separated from peers, suffered distress over A‐level assessment processes and an abrupt end to school education without the usual formal and celebratory closure. This cohort began a new educational chapter confused, uncertain and anxious and initially unprepared. Tensions during the first unit between tutor guidance and student autonomy were subsumed by pedagogies of care, with increased emphasis on developing relationships and trust. More time was dedicated to 1:1 and small group work to identify where additional tutor and peer support was needed. This approach helped students to rebuild everyday confidence and resilience, work with new tutors and peers, and subsequently engage in meaningful creative exploration.

### The impact of COVID‐19 on the spatial and material aspects of learning

During Unit 1, students would usually work in tutor groups around large studio tables, changing studios as they progressed through each discipline. These are ordinarily liminal spaces through which students pass to explore each discipline, thresholds to discovering an affinity with one creative space. The physical spaces and studios on the programme are designed to nurture creative practices through socially situated learning, where students learn by doing with ongoing opportunities for discursive collaboration. Ordinarily, all rooms allow for freedom of movement around desks, tables and fine art spaces so that learning does not occur in isolation. Moreover, movement is actively encouraged to facilitate iterative exchanges and sharing of knowledge so that learning is *“not merely situated in practice”* (Lave & Wenger 1991, p. 35). This approach builds on initial legitimate peripheral participation in its holistic sense where individuals progressively move towards *“more intensive participation”* (ibid, 1991, p. 36). As creative identities evolve and confidence grows, movement and use of space become more dynamic.

Social distancing rules during Covid meant that most studios resembled exam halls with individual desks in regimented rows. Unit 1 was delivered with tutor groups remaining in the same studio and seats throughout, with tutors moving studios for delivery. Students were denied the opportunity to develop familiarity with individual studio spaces across disciplines where much comfort, and therefore confidence, can be derived from a sense of space and place. These restrictions also had a noticeable impact on the development of new relationships, where students in previous years embrace this stage to form new bonds with like‐minded others. New peer relationships usually support a growing sense of stability for students, yet this year the lack of flexible studio access and social learning appeared to increase the need for pastoral support.

The regimented studio layouts and restrictions on movement over the past year presented significant challenges to maintaining the usual dynamic momentum, with intermittent lockdowns interrupting progression further. These aspects of the learning journey were beyond our control, and whilst the pandemic permeated all areas of education, creative courses in particular suffered. Creative students require access to other ‘making’ spaces, such as workshops, darkrooms and print rooms and are ordinarily offered hands‐on practical sessions to develop knowledge, skills and understanding of processes and equipment such as sewing machines, 3D printers, laser‐cutters, cameras and lighting. These spaces and resources, ordinarily, support the development of an open mind to new media, materials and methods; however, these rich learning spaces have been severely restricted for NCL‐FAD students this year.

During intermittent lockdowns and an emerging hybrid approach to delivery, digital technologies and platforms such as Microsoft Teams were quickly adopted as solutions to both curriculum delivery and communication. However, as a team, we were acutely aware that intermittent home‐based ‘studio practice’ would not truly replicate the practical and social experiences usually gained on campus. Moreover, we recognised that creative work produced from home would potentially be limited in terms of practical and material exploration. This also meant that areas such as Fine Art, where large‐scale canvases and sculptural media are widely used, were perhaps more disadvantaged than other areas such as Visual Communication and Fashion Communication, where digital technologies and outcomes are more accessible and to some extent expected.

**Figure 1:**
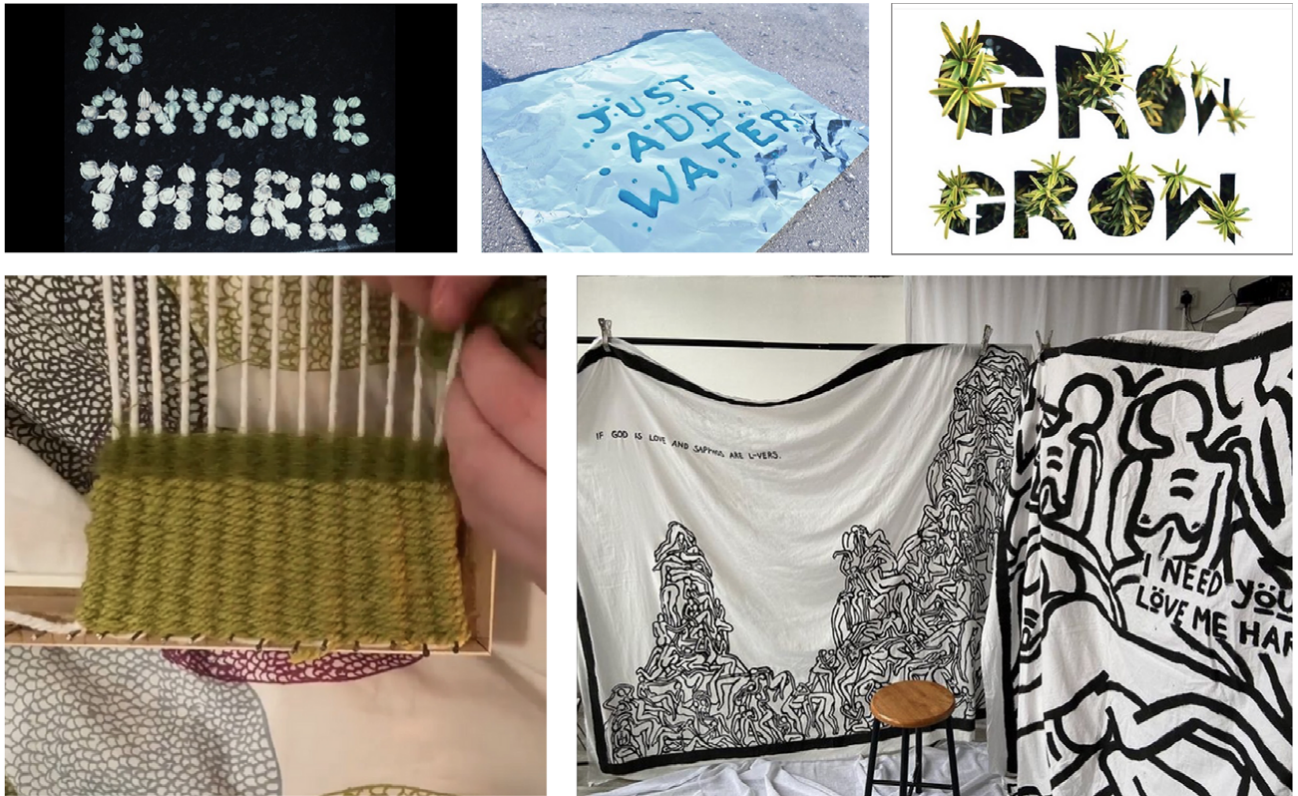
Student Experimentation with Media, Methods and Materials. [Colour figure can be viewed at wileyonlinelibrary.com]

Responding to the Covid situation, we placed more emphasis on process over outcome to encourage experimentation with a broad range of ‘found’ media and materials and available technologies. This encouraged students to become more open to a range of concepts and ideas alongside practical experimentation. In this respect, they were learning to ‘be’ artists and designers through new knowledge and research efforts, rather than learning to ‘do’ art and design by focusing on outcomes and artefacts (Taboada & Coombs 2013). The NCL‐FAD team deemed this approach highly successful, with students responding positively and embracing the new and often unexpected opportunities this allowed them to explore. Figure 1 (below) demonstrates student experimentation with imagery and text, handmade looms and unwanted bedding as canvases.

Encouraging students to consider multiple outcomes rather than work through linear processes towards one final piece resulted in work displaying ‘playfulness’ with media and technique and an openness to considering multiple perspectives for outcomes. Students began to understand the creative process as iterative, exploratory and sometimes chaotic (Razzouk & Shute 2012).

Moving image was adopted extensively this year as NCL‐FAD students explored immersive and authentic ways to communicate ideas with restricted resources, using outside spaces extensively in their work. This medium offered an opportunity to use everyday tools, such as smartphones, to plan, produce and edit creative ideas. As technology continues to advance refined ways of capturing the visual image through accessible tools, artists are now less restricted to using industry‐standard hardware and software, and whilst final outcomes were not always refined, the range of short films produced was effective in communicating concepts and narratives. Developing multi‐modal approaches to work across visual arts, it seemed that students were highly focused on meaning‐making by exploring media and resources which supported the authenticity, authority and reliability of the messages they were trying to convey.

Considering this new‐found openness to media, materials and methods as a threshold concept (Cousin 2006; Meyer & Land 2003), the experimental approach is something which affects both ontological and epistemological changes and is therefore *transformative*. The experience is *irreversible* and once understood opens opportunities for new experimental processes. In this sense, it is also *integrative* as students recognise how this applies to future work – allowing students to synthesise process and outcome. Experimenting with media, materials and methods is an integral component of creative pedagogy and therefore *bounded*. However, considering this characteristic as a conceptual learning space rather than delineating it as a form of ‘disciplinary property’ (Cousin 2006), allows students to recognise there are multiple ways to solve problems. The latter also reflects the *troublesome* characteristic in that there is ambiguity and uncertainty where there are multiple perspectives with no right or wrong answers. Whilst some experimentation with media, materials and methods was naïve and literal, these are still small victories in creative experimentation and represent a heuristic approach to learning.

### The impact of COVID‐19 on social and collaborative learning

Beyond the spatial and material aspects creative learning, these spaces would ordinarily facilitate a wealth of student engagement and cognitive development through social and collaborative learning. As learning in the visual arts is traditionally delivered through ongoing dialogue between tutors and students (Shreeve *et al*. 2010), it is both interactive and collaborative (Orthel & Day 2016; Page *et al*. 2006). Opportunities for learning emerge through formal and informal conversations between student and tutor, and equally between students. This communication and knowledge transfer is a key aspect of developing creative ‘communities of practice’ (Wenger 2000) whereby the open‐ended nature of creative outcomes is supported through the ongoing exchange of ideas and perspectives. The spatial restrictions this year prevented much of this collaboration. Tutor to student exchange appeared more didactic in approach due to restrictions on staff circulating rooms. Two studios represented ‘exam‐like’ layouts with one student per table; two studios were permitted two students per large table; and the movable panels in the fine art studio were positioned so that each student had a contained space between two walls. These layouts had a very isolating effect on students, conversations were tense and proved limiting to developing the usual sense of camaraderie.

In addition to studio conversations, a more formal approach to learning is established through studio critique with students sharing work for critical response. This aspect of creative pedagogy offers a formative assessment opportunity to observe students developing creative identities through dynamic exchanges of student voice and feedback. Models of studio critique take many forms, yet fundamental to the process is that individual work is justified and shared with peers and tutors, enabling self‐reflection and self‐evaluation based on alternative perspectives. The critique is an emotional and vulnerable space where students require confidence to speak about their work and resilience to accept the views and opinions of others – these are *troublesome* and new liminal spaces of uncertainty to be negotiated on NCL‐FAD.

**Figure 2:**
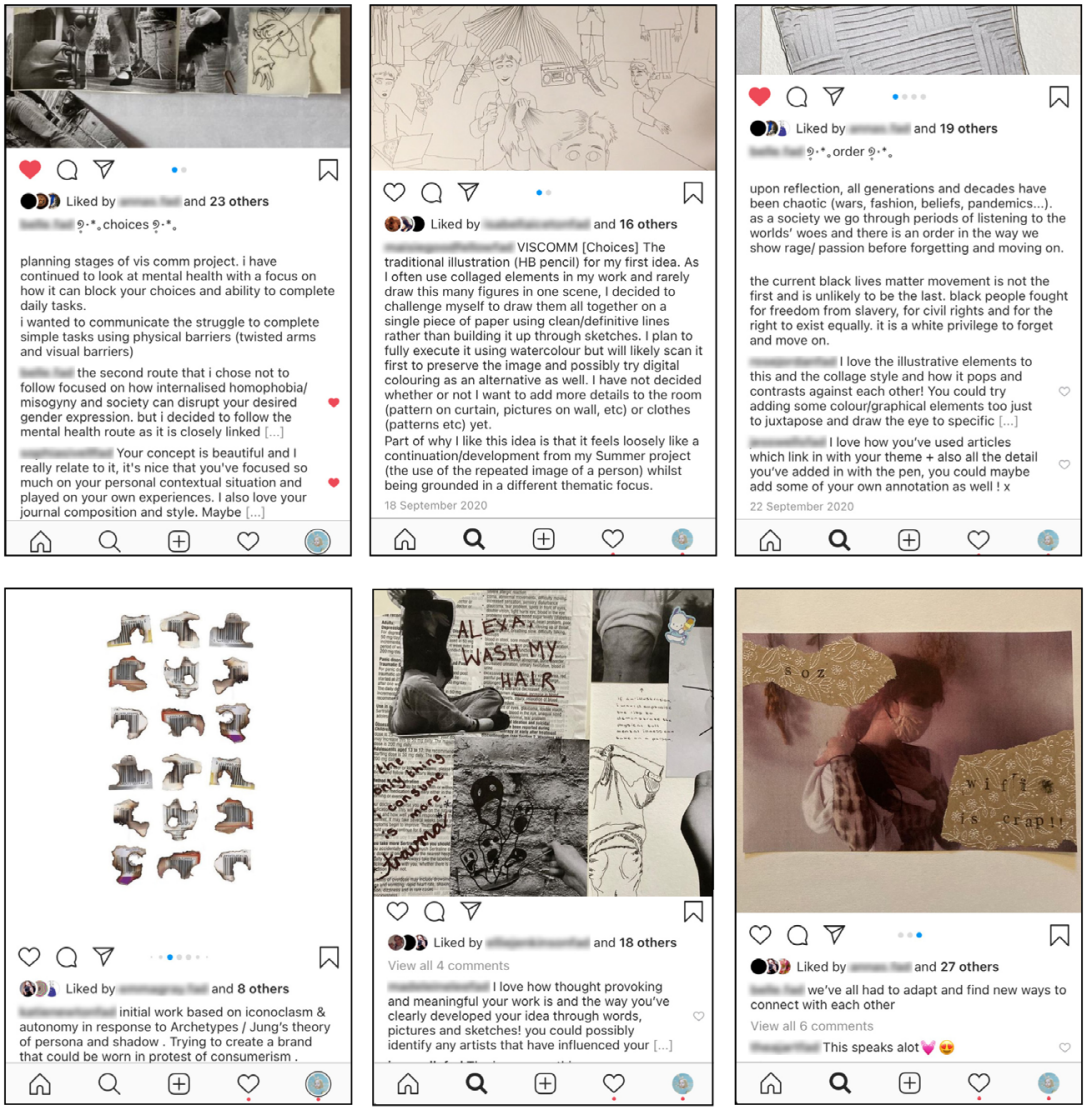
Developing a Feedback Loop and Validation through Digital Critique. [Colour figure can be viewed at wileyonlinelibrary.com]

Ordinarily, critiques are a regular occurrence, particularly during the second and third units of the programme. Yet this year, the physical spaces and restrictions proved difficult to navigate therefore critique required a different approach. As a team, we opted to use Instagram as a perceived familiar sharing platform, with new NCL‐FAD profiles only visible to staff and peers. This approach quickly proved to be of significant value for staff and students alike (see Figure 2 below). Students embraced this safe space for communication and emerging discussion was perhaps richer than previously achieved in live studio critique at this stage of NCL‐FAD. For students who struggle considerably with presenting work in the physical realm, this digital platform gave them a creative voice. Much of the commentary was insightful and meaningful, and this provided valuable validation as a significant aspect of the feedback loop (Botella *et al*. 2011), contributing to the development of creative confidence. For some visual arts educators, the speed of ‘networked knowledge’ and connective experiences render studio‐based learning less essential (Dreamson in Fleischmann 2020), and whilst debate around this is ongoing, there is no doubt that digital networks enabled valuable and accessible learning opportunities for the NCL‐FAD cohort.

Following the success of digital critique, we progressed to live online critique using Microsoft Teams in pairs and small groups. Initially, there were tensions around this and attendance proved challenging. However, with perseverance, students began to engage with this digital space sharing work on screen or via the chat function with real‐time discussion. As the NCL‐FAD programme progressed, students began to accept this new Teams approach as the ‘norm’, with confident students initiating their own peer reviews without tutor prompts. This suggests a changed mindset where students recognised the value of critique and alternative perspectives within the creative process, adopting this as an integral part of their own creative practice. As the student comments below demonstrate, the digital approach this year contributed to the positive and transformative experiences the NCL‐FAD tutor team aim for:

The use of Instagram and consistent critiques with peers helped me become less of an isolated worker and mix with lovely like‐minded people, (NCL‐FAD art student, 2021).

Being able to openly talk with my peers and bounce ideas back and forth is something I haven't had with past work… This on its own has made my creative process more effective, (NCL‐FAD design student, 2021).

### The impact of COVID‐19 on creative outcomes

Developing new and innovative ideas often proves problematic for NCL‐FAD students, where previous education has focused on the development of practical and technical skills over the cognitive skills required to think differently and challenge existing and pre‐conceived ideas. Yet over this year, students have clearly engaged with their emotions and experiences to develop creative work. Emotion has a significant impact on creativity (Botella *et al*. 2011) and the trauma, anxieties and experiences of students this year contributed to a broad range of globally aware, culturally conscious and socially oriented concepts. A significant number of projects responded to timely issues such as mental health, social isolation, climate change, immigration, sustainability, neurodiversity and hidden disabilities.

**Figure 3:**
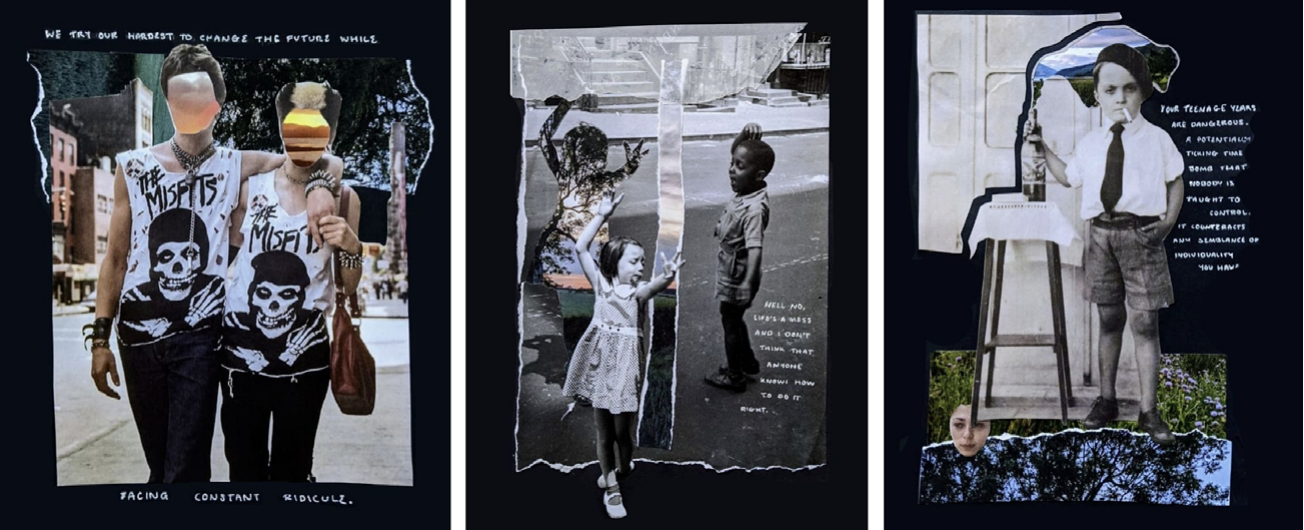
Emotion and Experience has Contributed to Diverse Concepts and Ideas. [Colour figure can be viewed at wileyonlinelibrary.com]

Student engagement with personal emotion often resulted in an extensive outpouring of concerns and fears within creative journals, and a stream of consciousness contributing to and supporting the development of meaningful outcomes. There is a sense that this NCL‐FAD cohort may perhaps have benefitted to some extent from an unprecedented global pandemic, where social isolation has allowed time for reflection and absorption of global events. Students have used this experience to inform creative output, responding to real‐world events for the final self‐directed unit, demonstrating empathy and understanding. For some, these creative responses have been coping mechanisms, an intuitive interrogation of perceived problems likely to remain for some time. This reflexive approach to real situations builds creative capacity and the ability to problem solve through a socially oriented praxis. The experience and impact of COVID‐19 on the NCL‐FAD cohort this year have played a significant role in informing a diverse range of creative ideas in response to personal and collective experiences (Figure 3).

### The impact of COVID‐19 on student progression

Informed student progression is a key aspect of the NCL‐FAD programme, which acts as a ‘signposted bridge’ to guide and support choices. The first unit of the programme helps students to develop an understanding of the creative disciplines available, with the second unit supporting further contextualisation in specialist areas to identify more specific pathways for progression. In this respect, the NCL‐FAD programme fulfilled its objectives this year to ensure that students explored progression options fully informed. Through both online and studio delivery, students were supported to identify individual strengths and develop a creative practice appropriate to chosen progression routes.

Portfolio preparation in previous years has often been challenging with early submission deadlines preventing the inclusion of work produced during later stages of specialism demonstrating new creative strengths. Photographing, editing, cropping and printing work for physical portfolios is time‐consuming and costly. Alongside this, the formatting of work for additional digital portfolios has always been problematic due to a lack of standardisation across universities for the formatting and submission of digital imagery. This year, however, all portfolios were digital and whilst standardisation was still lacking, there were significant benefits in this approach. Firstly, this cut out the significant cost of buying physical portfolios and large scale printing. Secondly, through both in person and online (synchronous and asynchronous) communication, the process allowed flexibility to plan, discuss and review portfolios effectively and efficiently. Whilst the digital portfolios did not support the sharing of extensive bodies of work, following specific content guidance the work was strong and demonstrated student skills and abilities effectively.

Although NCL‐FAD faced a year of challenges due to Covid restrictions, this cohort continued to reflect the successes of previous NCL‐FAD student progression. All students applying through UCAS were offered places on degree programmes, with the majority securing offers from first choice universities. Each year, a small minority of students choose not to progress to HE, with some seeking apprenticeships where possible or taking a ‘gap year’. This year, approximately six from a cohort of 87 students chose not to pursue an undergraduate degree, reflecting similar numbers to previous years. From this data, it would seem that the NCL‐FAD programme succeeded in fulfilling its progression purpose despite Covid restrictions.

Finally, in terms of further success, 32 per cent of the cohort on the NCL‐FAD programme this year achieved Distinction grades, a higher proportion than in previous years. Whilst the grading process this year allowed a more holistic approach, the level of critical and conceptual engagement was clearly evident during the assessment. A robust approach to double‐marking supported the team to review and clarify consistent grading, also affording further insight into the work produced across each discipline to confirm the strength of work across the cohort. When informally questioned on ‘prepared‐ness’ for HE, NCL‐FAD student feedback was positive, reinforcing the notion of a post‐liminal stage with a clear sense of self and new creative identities:

The support was fluid when transitioning to and from online learning, but this course gave me the much needed independence in creating and developing ideas so that I feel much more ready for the transition to university, (NCL‐FAD art student, 2021).

This year has helped me develop skills specific to my progression in graphic design, develop a strong portfolio and gain a place at my first choice university course, (NCL‐FAD design student, 2021).

## CONCLUSION

This article has reflected on the learning journey of an NCL‐FAD cohort of students during the ongoing disruption of COVID‐19. Using van Gennep’s (1909) three stage process to consider this year as a liminal and transformative learning journey, the NCL‐FAD model has been framed as a *‘Rite of Passage’* for art and design students whose goal is to progress to undergraduate degree programmes. Discussion has considered the pre‐liminal stage where signs of trauma emerged at the beginning of the programme. As a result, tensions during the first unit marked a shift in focus towards tutor‐guided competencies and pedagogies of care, with an emphasis on pastoral support. Working closely with small groups and paired students, the development of relationships and the re‐building of confidence allowed this NCL‐FAD cohort to engage in creative exploration.

During the second unit, and despite the ongoing uncertainties of COVID‐19, student confidence began to flourish. Discussion identified how students adapted to online learning and adopted an openness to home studios by exploring media, methods and materials for work. Student engagement during this liminal stage was further enhanced when the cohort responded proactively to Instagram as a platform for critique and subsequently accepted new digital spaces as the ‘norm’. This aspect of the transformative journey demonstrated how students moved into the post‐liminal stage, initiating self‐directed peer critiques clearly suggested the incorporation, internalisation and acceptance of new norms and values. The use of digital tools during 2020–21 offered an effective temporary solution to ongoing disruption, however, this is not to say that this can be a long‐term alternative for face‐to‐face teaching. Just as flood communities in environmental crises use social spaces such as leisure centres for temporary shelter, a return home is always welcome and so it is with a return to physical studios.

In considering whether the NCL‐FAD programme fulfilled its aims and objectives over this difficult year, it appears that the combination of successful physical and tangible outcomes over the year have blended effectively with the emotional and intellectual outcomes to produce a cohort of students ready to ‘be’ artists and designers. They have demonstrated the emotional resilience and emotional confidence to support their progression to HE where the uncertainties of COVID‐19 still linger.
